# To be, or not to be biodegradable… that is the question for the bio‐based plastics

**DOI:** 10.1111/1751-7915.12393

**Published:** 2016-08-01

**Authors:** Auxiliadora Prieto

**Affiliations:** ^1^ Polymer Biotechnology LabCentro de Investigaciones Biologicas (CIB)C/Ramiro de Maeztu, 928040MadridSpain

## Abstract

Global warming, market and production capacity are being the key drivers for selecting the main players for the next decades in the market of bio‐based plastics. The drop‐in bio‐based polymers such as the bio‐based polyethylene terephtalate (PET) or polyethylene (PE), chemically identical to their petrochemical counterparts but having a component of biological origin, are in the top of the list. They are followed by new polymers such as PHA and PLA with a significant market growth rate since 2014 with projections to 2020. Research will provide improved strains designed through synthetic and systems biology approaches; furthermore, the use of low‐cost substrates will contribute to the widespread application of these bio‐ based polymers. The durability of plastics is not considered anymore as a virtue, and interesting bioprospecting strategies to isolate microorganisms for assimilating the recalcitrant plastics will pave the way for in vivo strategies for plastic mineralization. In this context, waste management of bio‐based plastic will be one of the most important issues in the near future in terms of the circular economy. There is a clear need for standardized labelling and sorting instructions, which should be regulated in a coordinated way by policymakers and material producers.

## Global warming, market and production capacity, the key drivers for selecting the main players

The unparalleled success of plastics shows no signs of slowing down, and total plastics consumption could grow from the current 250 000 kilotonnes per year to about 1 million kilotonnes by the end of this century. The Intergovernmental Panel on Climate Change trajectory to 2050 for stabilization of atmospheric greenhouse gas (GHG) concentrations at 450 ppm CO_2_ requires an 80% reduction in emissions compared to the 1990 level (http://www.ipcc.ch/publications_and_data/ar4/wg3/en/contents.html and http://unfccc.int/paris_agreement/items/9485.php). The vast majority of plastics in current production are derived from crude oil, thus, besides the resistance to biodegradability, their GHG emissions are an environmental issue. Considering climate change as a grand societal challenge, establishing a market of bio‐based plastics is seen as virtuous in terms of Bio‐economy, due to their bio‐based carbon content (Philp *et al*., 2013).

Consequently, the demand for bio‐based polymers is growing worldwide. The chemical industry is continuing to produce traditional plastics but, in parallel, it is developing a broad range of bio‐based, biodegradable or non‐biodegradable, and/or compostable plastics. In this scenario, we should distinguish between two main groups of players; on the one hand, the new bioplastics based on renewable resources, either monomers or full biopolymers. Some of these are biodegradable plastics and are produced by fermentation or chemical processes, while others are produced by a combination of biotechnological and chemical processes. Examples are polyhydroxyalkanotes (PHA), polylactic acid (PLA), succinic acid and 1,3‐propanediol based polymers, etc. The second group comprises the drop‐in bio‐based polymers, chemically identical to their petrochemical counterparts but they have, at least partially, a component of biological origin such as the bio‐based polyethylene terephtalate (PET) or polyethylene (PE). The term drop‐in was initially used for biofuels whose specifications allow market applications with existing infrastructure and avoid important investments. Drop‐in plastics are non‐biodegradable materials, obtained from renewable raw materials that present identical technical properties to their fossil counterparts.

By 2019, worldwide production capacity of bio‐based plastics will grow by over 400%, or from 1.7 million tonnes in 2014 to 7.8 million tonnes in 2019 in absolute terms. The market is clearly being dominated by drop‐in bio‐based polymers and non‐biodegradable polymers. Drop‐in bio‐based polymers such as PET and PE lead this category. Bio‐based PET is the overall market leader and is expected to grow quickly, from 35.4% in 2014 to 76.5% in 2019. Consequently, the bio‐based non‐biodegradable polymers market is expected to grow strongly. PET production capacity was around 600 000 tonnes in 2014 and is projected to reach about 7 million tonnes by 2020, using bio‐ethanol from renewable sources. PET is currently 20% bio‐based and produced from bio‐based monoethylene glycol and terephthalic acid (TPA). The huge bio‐based market demand is mainly the result of the ‘Plant PET Technology Collaborative’ comprising Coca Cola, Ford, Heinz, Nike and Proctor & Gamble (http://www.plasticstoday.com/five-major-us-brands-collaborating-plant-based-pet/69477799417544). This consortium and other retailers are assisting several pre‐commercial enterprises to develop commercial processes for bio‐based TPA, which is currently still petro‐based but subject to ongoing research. Most of these processes target p‐xylene, the petrochemical precursor for TPA (Smith, 2015).

Recently, PET 100% bio‐based polymer alternatives with potentially similar applications based on furanic building blocks are being developed such as Poly‐ethylene‐furanoate (PEF) and polytrimethylene furandicarboxylate (PTF). Avantium patented the technology to convert biomass into FDCA (2,5‐Furandicarboxylic acid), which is the monomer used to produce the bio‐based polyester PEF. DuPont and ADM plan to make PTF by reacting fructose‐derived furandicarboxylic methyl ester with 1,3‐propanediol (https://cen.acs.org/articles/94/i12/BASF-Avantium-combine-bio-polyester.html).

Exact figures for worldwide biodegradable bioplastics production and capacities are difficult to determine. They are mostly based on estimates, and these are constantly changing due to the rapid growth of the bioplastics industry. According to the study of the Nova‐Institute (http://www.bio-based.eu/market_study/media/files/15-12-03-Bio-based-Building-Blocks-and-Polymers-in-the-World-short-version.pdf), following the bio‐based drop‐in polymers, PHA shows the second fastest market growth rate since 2014 with projections to 2020. PHA production capacity was around 35 000 tonnes in 2014 and is projected to reach about 100 000 tonnes by 2020. PHAs are biodegradable biocompatible polyesters, which accumulate as granules in the cytoplasm of many bacteria under unbalanced growth conditions. PHA diversity (recently described as ‘PHAome’) has increased rapidly due to the increasing diversity of monomers, homopolymers, random and block copolymers, functional and graft polymers, molecular weights and combinations of the above. Moreover, successful manipulation of β‐oxidation in *Pseudomonas* spp. has provided control over the above diversity (Chen *et al*., 2015). These species produce mainly medium‐chain‐length PHAs (mcl‐PHAs), which are promising thermo‐elastomers because they can be further modified by inserting functional groups in the side‐chains. Functionalized mcl‐PHAs provide modified mechanical and thermal properties, and consequently have new processing requirements and highly diverse potential applications in emergent fields such as biomedicine. However, process development and sample availability are still limited due to the toxicity of some precursors and current low productivity, which hinder investigation. Conversely, improved mutant strains designed through systems biology approaches and co‐feeding strategies with low‐cost substrates may contribute to the widespread application of these biopolymers (Prieto *et al*., 2016).

Growth rates of polybutylene succinate and PLA are also impressive; their production capacities are expected to increase very significantly between 2014 and 2020. PLA production capacity was around 200 000 tonnes in 2014 and is projected to reach about 450 000 tonnes by 2020. PLA is used for a broad spectrum of applications, ranging from fibre products to bottles and packaging foams. PLA can not only be used in its pure form but also several derived co‐polyesters make up the core of the biodegradable polymer markets as disposable, short‐life products. Thin‐walled goods bags or bio‐waste make up two‐thirds of the total consumption today and this share is expected to grow further until 2020 (Castro‐Aguirre *et al*., 2016).

Finally, starch‐based materials are also pushing into new markets like, for example, coffee capsules or aquaculture items. Functional products such as barrier packaging and various biodegradable outdoor uses are currently low volume but have a significant potential for market breakthrough.

Over the past decade, we have gained a better understanding of the molecular mechanisms and regulatory processes underlying the biotechnological synthesis of bio‐based polymers and monomers. Knowledge in this field is the foundation for metabolic‐ and protein‐engineering approaches to improve economic‐production efficiency and produce tailor‐made polymers with highly applicable material properties (Chung *et al*., 2015).

## Biodegradability versus durability and the issue of waste management

The durability of plastics was originally viewed as a virtue; however, this durability has created environmental problems, and led to the early research and development of the first biodegradable plastics. Pure aromatic polyesters like PET are traditionally considered as quite insensitive to any hydrolytic degradation. Under drastic chemical conditions (e.g. sulphuric acid at 150°C) hydrolysis of such polyesters can be used for recycling purposes.

Due to the requirement of these energy‐consuming conditions for chemical recycling, the application of polyester hydrolases has been proposed as an environmentally friendly alternative for PET recycling (Wei *et al*., 2014). Polyester hydrolases from several fungi and bacteria have already been evaluated for their activity against PET fibre surfaces with applications in the textile and detergent industries. Efficient hydrolysis of synthetic polyesters requires reaction temperatures close to their glass transition temperature (Tg) (approximately 75°C for PET). Thermophilic microorganisms are therefore an interesting source of thermostable hydrolases capable of degrading aromatic polyesters. All polyester hydrolases from actinomycetes described so far are members of the α/β hydrolase fold superfamily of enzymes, similar to the well‐known PHA depolymerases for the degradation of PHA (Knoll *et al*., 2009). In the past decade, a growing number of enzymes capable of hydrolysing synthetic polyesters have been identified from thermophilic actinomycetes of *Thermobifida* and *Thermomonospora* species (Wei *et al*., 2014). They demonstrate versatile hydrolytic activity against both soluble and insoluble substrates, including PET and other aromatic polyesters. Their thermal stability at temperatures between 50 and 60°C make these enzymes applicable to the surface modification of polyester films and fibres. However, we still require more detailed knowledge of the factors influencing thermal stability and substrate binding to engineer polyester hydrolases with a superior ability to hydrolyse synthetic polyesters, opening up new avenues for protein evolution using rational design strategies. Microbial cutinases and enzymes with applications to surface modification of PET also play a key role in this field. To develop enzymes with further enhanced activity will require a better understanding of the interaction of the enzyme with the substrate regarding factors such as sorption, movement on the polymer surface, and the role of hydrophobins or binding modules (Guebitz and Cavaco‐Paulo, 2008).

Very recently, a mesophilic bacterium that degrades and assimilates PET has been isolated by Yoshida *et al*. (2016). These authors screened natural microbial communities exposed to PET in the environment, and thus isolated a novel bacterium, *Ideonella sakaiensis* 201‐F6, which is able to use PET as its major energy and carbon source. When grown on PET, this strain produces two enzymes capable of hydrolysing PET and the reaction intermediary, mono(2‐hydroxyethyl) TPA. Both enzymes are required to enzymatically convert PET efficiently into its two environmentally benign monomers, TPA and ethylene glycol. These are interesting bioprospecting strategies to isolate microorganisms for assimilating these recalcitrant plastics and, therefore, pave the way for *in vivo* strategies for plastic mineralization. In this context, plastics are the most abundant form of marine debris, with documented impacts on some marine environments, although the influence of plastic on microbial communities in ocean ecosystems is poorly understood. Amaral‐Zettler and her co‐workers collected plastic marine debris at multiple locations in the North Atlantic and analysed the attached microbial communities. They found a diverse microbial community of heterotrophs, autotrophs, predators and symbionts; a community that they referred to as the ‘Plastisphere’ (Zettler *et al*., 2013). These findings highlight the potential niche for plastic‐degrading bioprospecting strategies.

In contrast to drop‐in plastics, biopolymers like PHA are, by definition, biodegradable, and so their application as commodity products becomes increasingly attractive in view of the need to avoid recalcitrant oil‐based polymers. When exposed to the microbial flora present in a given environment (e.g. in soil or water), biopolymers are fully degraded and mineralized to CO_2_ and H_2_O. Secreted depolymerases and hydrolases attack the biopolymer backbone, leading to low‐molecular‐mass degradation products, which can then be taken up by the microbial cell and used as carbon and energy sources (Knoll *et al*., 2009). The biodegradability of these materials has been widely demonstrated at lab scale but the biodegradation of these upcoming bioplastics (bio‐based polymers and their blends) needs to be demonstrated at pilot scale in waste management plants.

Research shows PLA is absorbed in animals and humans and, hence, it is extensively used in biomedicine. The degradation of the polymer in animals and humans is thought to occur via non‐enzymatic hydrolysis. Several enzymes can degrade the polymer, including proteinase K, pronase and bromelain. However, few have been characterized with regard to microbial degradation of the polymer. PLA is also readily degraded in compost (Castro‐Aguirre *et al*., 2016).

Waste management of bio‐based plastic will be one of the most important issues in terms of the circular economy. In an ideal 2020 city scenario, in which the bioplastic would be subject to sorting instructions, biodegradable plastics could either be collected with other packaging, residual waste or with organic waste. However, any of these waste management channels could have drawbacks, either during the separation process at origin and/or in the biodegrading process in waste management plants. Biodegradable plastics and their blends are not always compostable plastics; biodegradable plastics are mineralizable due to the action of microorganisms and enzymes. Through this process, the materials are converted into carbon dioxide, methane, water and biomass. However, compostable plastics conform to the official standards according to EN 13432, which specifies they must be compostable only under specific conditions (temperature, humidity level, time) in the composting system, and should lack toxic side‐effects for water, soil, plants or living organisms. To deal with this standardization in the biodegradation process, scientists should address composting solutions based on microbial communities with complementary hydrolytic activities for a broad substrate range of bio‐based polymers and their additives. Otherwise, the advantages of biodegradable plastics will be futile. Concerning the separation of waste at origin, the different labels indicating how the bioplastics should be collected and sorted might be extremely confusing for the consumer. There is a clear need for standardized labelling and sorting instructions, which should be regulated in a coordinated way by policymakers and material producers (more information at http://www.pro-e.org/Fact-sheet-on-bioplastics.html).

## Unexploited feedstock of international origin for bio‐based plastics production

It is widely accepted that the price of the carbon source is one of the main factors affecting the cost of bio‐based plastics, influencing the sustainability of production processes. However, the choice of a suitable carbon source is not a clear‐cut issue and there is debate around the merits of using plant‐based biomass normally used as a food source for the production of non‐food chemical products, such as fuels. This debate has promoted the search for other sources, such as the use of waste. There are some bio‐wastes that share more or less homogeneous compositions (e.g. glycerol, vegetal oils, corn steep liquor, molasses, milk, meat), which can be managed using various biological processes. The use of industrial and agricultural by‐products could require extensive purification, equalling or even surpassing the energy demand of cost‐intensive agricultural feed‐stocks. Also, as waste complexity increases, so too does their disposal and/or recycling, requiring the integration of different technologies to achieve more efficient processes. In the particular case of bio‐wastes, technologies are integrated through the concept of bio‐refinery. In fact, the production of bio‐based and biodegradable polymers from renewable resources is an area of intense research and industrial activity (Koutinas *et al*., 2014).

However, a significant portion of waste is poorly biodegradable and cannot be easily converted into new added‐value chemicals or polymers by microorganisms or other biological processes because it contains mixtures of very complex compounds and some toxic pollutants, which are highly recalcitrant to degradation. In this context, the use of plastic wastes has recently been proposed as a novel second‐generation carbon source for biotechnology, supported by the analogy to the mega‐developments in lignocellulosic biotech. This idea is supported by the fact that carbon‐rich polymers have a relatively simple and well‐defined composition compared with biomass, and they are also extremely abundant, which will empower the recycling industry to a qualitatively new dimension (Wierckx *et al*., 2015). In 2010, plastics comprised 12.4% (31 million tonnes) of total municipal solid waste in the United States (United States Environmental Protection Agency (USEPA), 2011). Only 7.6% of that plastic waste was recovered for recycling, leaving 28.7 million tonnes of plastic for landfill or incineration. In Europe, 25 million tonnes of plastic waste is generated yearly, of which some 60–80% is managed in this way with the remaining 20–40% being materially recycled (http://www.plasticseurope.org/documents/document/20150227150049-final_plastics_the_facts_2014_2015_260215.pdf). Although still in their infancy, bioprospecting strategies are being developed to isolate microorganisms able to assimilate recalcitrant plastics; this will yield potential drivers to design bio‐based up‐cycling activities from plastic wastes.

Another example of insufficiently exploited bio‐wastes can be found in the municipal and commercial wastes and the sludge derived from urban water treatment. These potential raw materials contain significant reusable carbon fractions that are suitable for revalorization processes. According to the European Energy Agency, the use of municipal waste as a resource could cut GHG emissions by 62 million tonnes of carbon dioxide equivalents by 2020 compared with 2008 (European Environment Agency (EEA), 2011). Thermochemical conversion techniques, other than incineration/combustion, such as gasification and pyrolysis are becoming widely accepted as suitable alternatives. These gasification processes can convert any carbonaceous material into a synthesis gas (or syngas), which is predominantly composed of hydrogen, carbon monoxide and carbon dioxide. Syngas can serve directly as a fuel in gas engines, or it can be used to produce hydrogen, methanol, converted to liquid fuels via Fischer–Tropsch reactions or applied in fermentation processes to produce biofuel, chemicals and bioplastics. Recently, gasification and pyrolisis of different waste streams has attracted scientific and industrial attention, including the use of petrochemical plastic waste to produce fermentable syngas. (Drzyzga *et al*., 2015). Research related to the fermentative production of chemicals from syngas has greatly increased in recent years, recognizing the potential of using biological means to convert CO and CO_2_ to chemicals. On the one hand, the drawbacks of this technology include low rates of bacterial cell growth, transference of CO and H_2_ from the gas phase to the liquid phase of the fermentation medium, and low rates of bioproduction. On the other hand, the advent of efficient and inexpensive methods to sequence complete genomes and omic‐technologies have shed light on the metabolic and regulatory pathways of several syngas‐fermenting microorganisms. Also concerning gas fermentation, one of the most challenging strategies is the production of biopolymers and other bio‐based products from solar energy and carbon dioxide by artificial photosynthesis, converting sunlight to electricity and H_2_ *via* water electrolysis. An autotrophic H_2_‐oxidizing bacterium fixes CO_2_ in dark conditions. The assimilated CO_2_ is stored in bacterial cells as PHA. Compared with natural photosynthesis of a fast‐growing cyanobacterium, the artificial photosynthetic system has much higher energy efficiency and greater yields of bio‐based products (Yu, 2014). Progress is underway in these kinds of multidisciplinary strategies, combined with the latest systems biology tools, which are being applied to these microorganisms to enhance polymer or monomer production from gaseous C1 compounds via genetic and metabolic modification. Meanwhile, methods such as medium optimization and reactor design are being pursued to enhance polymer production from existing and new CO/CO_2_‐fermenting microorganisms.

Finally, mixed microbial cultures have been identified as promising processes for the production of some monomers and PHA from certain waste, such as sludge from water‐treatment plants. This is because these systems are based on the use of open cultures, which can use a wide variety of complex nutrient‐rich substrates, and, as opposed to most pure cultures, PHA storage is not induced by nutrient limitation. This is particularly advantageous when using industrial feedstock waste containing compounds of undefined composition (Serafim *et al*., 2008). However, the possibility of using the facilities already existing in wastewater treatment plants to produce PHA should be explored and evaluated as a cost‐effective technology. The future of this technology might be envisioned with the synergistic development and integration of omic‐systems biology tools (including technologies for population profiling and analysis) and analytical tools for studying molecular interactions in microbial populations. The interaction mechanisms of complex mixed microbial cultures could be thoroughly explained and understood, and the rational engineering of these microbial communities might thus pave the way for a host of intriguing applications in bio‐waste revalorization to biopolymers.

## Concluding remarks

In summary, climate change, market demand and production capacity have selected drop‐in plastics as the leaders of the bio‐based polymers market. BioPET is set to become the bio‐based polymer with the biggest production capacity in upcoming years while biodegradable plastics like PHA and PLA will experiment an upward market trend until 2020. Research will provide improved strains designed through synthetic and systems biology approaches; furthermore, the use of low‐cost substrates will contribute to the widespread application of these biopolymers. The availability of high‐throughput experimental tools and quantitative analysis techniques currently facilitates the design of more robust metabolic engineering strategies aiming to enhance and/or tailor this bio‐based plastic/monomer production. Exploitation of complex bio‐wastes will support the development of a bio‐based plastic market. Durability of bio‐based drop‐in plastics may impede the up‐cycling of these materials in terms of circular economy and sustainability. Combined biodegradation and bioprospecting strategies have emerged to address this issue. In line with these developments, waste management of bio‐based plastics needs to be standardized and regulated by governmental policymakers.

## Conflict of interest

The authors declare no conflict of interest.
